# Efficacy of Exercise Interventions in Patients with Acute Leukemia: A Meta-Analysis

**DOI:** 10.1371/journal.pone.0159966

**Published:** 2016-07-27

**Authors:** Yuan Zhou, Jinjie Zhu, Zejuan Gu, Xiangguang Yin

**Affiliations:** 1 The First Hospital Affiliated with Nanjing Medical University, Nanjing, Jiangsu, PR China; 2 College of Aerospace Engineering, Nanjing University of Aeronautics and Astronautics, Nanjing, Jiangsu, PR China; 3 Department of Nursing, The First Hospital Affiliated with Nanjing Medical University, Nanjing, Jiangsu, PR China; 4 College of Nursing, Nanjing Medical University, Nanjing, Jiangsu, PR China; Universidad Europea de Madrid, SPAIN

## Abstract

**Background:**

Decreased physical performance and impaired physiological and psychological fitness have been reported in patients with acute leukemia (AL). We performed a meta-analysis to assess the efficacy of exercise in patients with AL.

**Methods:**

In this meta-analysis, the electronic databases MEDLINE, Embase, Cochrane, Web of Science, SPORTDiscus, CINAHL and PEDro were searched through November 2015. Three authors participated in the study selection, data extraction and quality assessment. The instrument used for quality assessment was derived from the Cochrane Handbook for Systematic Reviews of Interventions. Analyses were performed according to the recommendations of The Cochrane Collaboration using Review Manager 5.3.

**Results:**

Nine trials (8 randomized controlled trials and 1 quasi-experimental design trial) with 314 AL participants were included in this meta-analysis. The pooled standardized mean differences between the exercise and control groups were 0.45 (95% confidence interval (CI): 0.09 to 0.80, P value = 0.01, P for heterogeneity = 0.23, I^2^ = 28%) for cardiorespiratory fitness and 0.67 (95% CI: 0.28 to 1.06, P value = 0.0007, P for heterogeneity = 0.14, I^2^ = 43%) for muscle strength. Based on the data for fatigue, anxiety, and depression, there were no significant differences in these parameters between the exercise and control groups.

**Conclusions:**

Exercise has beneficial effects on cardiorespiratory fitness, muscle strength and functional mobility; however, no significant improvements in fatigue, anxiety, depression or quality of life were observed. Further large-scale randomized trials are needed to assess the safety, feasibility and efficacy of exercise programs for AL patients.

## Introduction

Cancer patients gradually exercise less as the disease progresses and typically get less than the recommended level of exercise, as stated in previously published studies [1,2]. This decline is caused by various factors, including cardiopulmonary, gastrointestinal and neurological toxicities, anemia, thrombocytopenia and cachexia [3]. Moreover, fatigue, anxiety, depression, fear and the risk of infection, along with the oncologist’s recommendation of prolonged rest, may further limit a patient’s exercise options [4]. A low level of exercise has been extensively reported to have negative impacts on cardiopulmonary and musculoskeletal function [5,6], cognitive abilities [7], social function, and psychological well-being [8]. Therefore, exercise has been implemented in breast cancer patients to improve physical function and activities of daily living since the 1980s [9]. Most subsequent studies have intensively investigated exercise in relation to colorectal and prostate cancers, in addition to other types of solid tumors [10–12]. As an ancillary treatment to oncotherapy, exercise may improve quality of life (QOL), physical fitness, mood and many other factors in patients with solid tumors [13]. For hematologic cancers, some evidence has suggested that exercise may improve oxygenation and physical well-being, in addition to increasing muscle strength and cardiorespiratory fitness [4,14–16]. Therefore, exercise has gradually become integrated into cancer treatment programs because of its positive impacts.

Acute leukemia (AL) is a malignant hyperplastic disease of the hematopoietic system that is associated with high morbidity and that typically develops rapidly. Cancer statistics from 2016 [17] showed that among the 26,540 estimated new cases of AL in the United States, 11,860 deaths occurred. AL patients may feel quite fatigued, bruise easily and develop frequent infections, which are the main factors resulting in low levels of exercise among this population. As stated previously, a low level of exercise negatively affects physical and psychological function. Therefore, the efficacy of exercise as a component in the treatment of AL patients must be assessed.

Through a literature search, we identified only one systematic review (SR) on the effects of exercise in leukemia patients [16]. It is a descriptive review that lacks aggregated data and includes both acute and chronic leukemia subjects. Notably, the pathogeneses, clinical manifestations, treatments and other aspects differ between these two types of leukemia, and these differences were not considered in that review. Moreover, differences in survival rates and therapies between adults and children were not taken into consideration [18]. With the considerable growth in this research area, exercise studies of AL patients [19–21] that are not included in existing reviews have recently been published. Here, we performed meta-analysis to critically assess the effects of exercise on cardiorespiratory fitness, muscle strength, fatigue, anxiety, and depression in AL patients. In addition, subgroup analyses were performed to evaluate the effects of exercise in adult/pediatric patients and in those receiving induction/post-remission therapy.

## Materials and Methods

### Inclusion criteria and search strategy

The following inclusion criteria were applied: (1) population: human participants with a diagnosis of AL (either acute myelocytic leukemia (AML) or acute lymphoblastic leukemia (ALL)) undergoing induction therapy or post-remission therapy; (2) intervention: an exercise component, regardless of the type of exercise; (3) comparison intervention: standard care with no exercise intervention or instruction; (4) outcome measures: cardiorespiratory fitness, muscle strength, fatigue, anxiety, depression, QOL, hemoglobin level, body mass index (BMI) and functional mobility; and (5) study design: a randomized controlled trial (RCT) or a quasi-experimental design trial. To maximize the number of eligible studies for review, no restrictions were placed on sample size or age. Subgroup analyses were conducted based on adults/children and induction/post-remission therapy.

In this meta-analysis, seven databases, including MEDLINE, Embase, Cochrane, Web of Science, SPORTDiscus, CINAHL and PEDro, were searched through November 2015. This computerized search was performed using the following terms: (leukem* OR leukaem*) *AND* (exercise OR exercise movement techniques OR training program* OR physiotherapy OR physical therap* modality OR physical endurance OR physical exertion OR physical fitness OR physical education and training OR physical activity OR walking OR exercise therapy OR rowing* OR ramble* OR rambling* OR danc* OR mountaineer* OR cycling*OR bicycling* OR swimming* OR jogging* OR ambulation* OR running* OR athletic* OR sport* OR stretching* OR yoga* OR tai chi OR tai ji OR ji quan tai OR train* OR pilates* OR calisthenic* OR gymnastic*) *AND* (*trial OR randomized OR clinical trials OR placebo OR randomized OR controlled clinical trial OR randomized controlled trial*). The search strategy combined text words with medical subject heading terms and was limited to human subjects. No language restriction was applied.

### Study selection and data extraction

Two authors (Zhou and Zhu) independently screened the retrieved results that met the inclusion criteria by reading the abstracts and titles. If any question arose as to whether a study should be included, the full text was searched. In cases of disagreement, a third author (GU) joined the discussion to reach a consensus.

For each study, the following data were extracted by the reviewer (Zhou): the study details (first author’s last name, year of publication, and type of study); participant characteristics (diagnosis, department, timing, sample size, age, gender, and location); intervention groups (frequency, intensity, time, type, and duration); comparison groups; outcome measured; follow-up times; and funding. All extracted data were verified by a second reviewer (Zhu). The extracted data was imported into a Microsoft Excel file, and the data were again verified by a third investigator (Gu). Any discrepancies were resolved by discussion until a consensus was reached.

### Quality assessment

The methodological quality and risk of bias of each trial were evaluated according to the Cochrane Handbook for Systematic Reviews of Interventions [22]. The following criteria were included: random sequence generation (selection bias); allocation concealment (selection bias); blinding of participants and personnel (performance bias); blinding of the outcome assessor (detection bias); incomplete outcome data (attrition bias); selective reporting (reporting bias); and other biases. The quality assessments were independently conducted (Zhou and Zhu), and any disagreements were resolved by discussion.

### Data analysis

The data from the included studies were sufficient for conducting meta-analysis. Analyses were performed according to the recommendations of the Cochrane Collaboration, and Cochrane Statistical Package Review Manager 5.3 was used for analysis. For the continuous outcomes, we calculated the mean difference (MD) or standardized mean difference (SMD) with the 95% confidence interval (CI) for each study. Study heterogeneity was assessed using the Chi² test at a significance level of P<0.1, in addition to the I² statistic (I²>50% indicated substantial heterogeneity) [22].

## Results

### Search results

**Fig 1:**
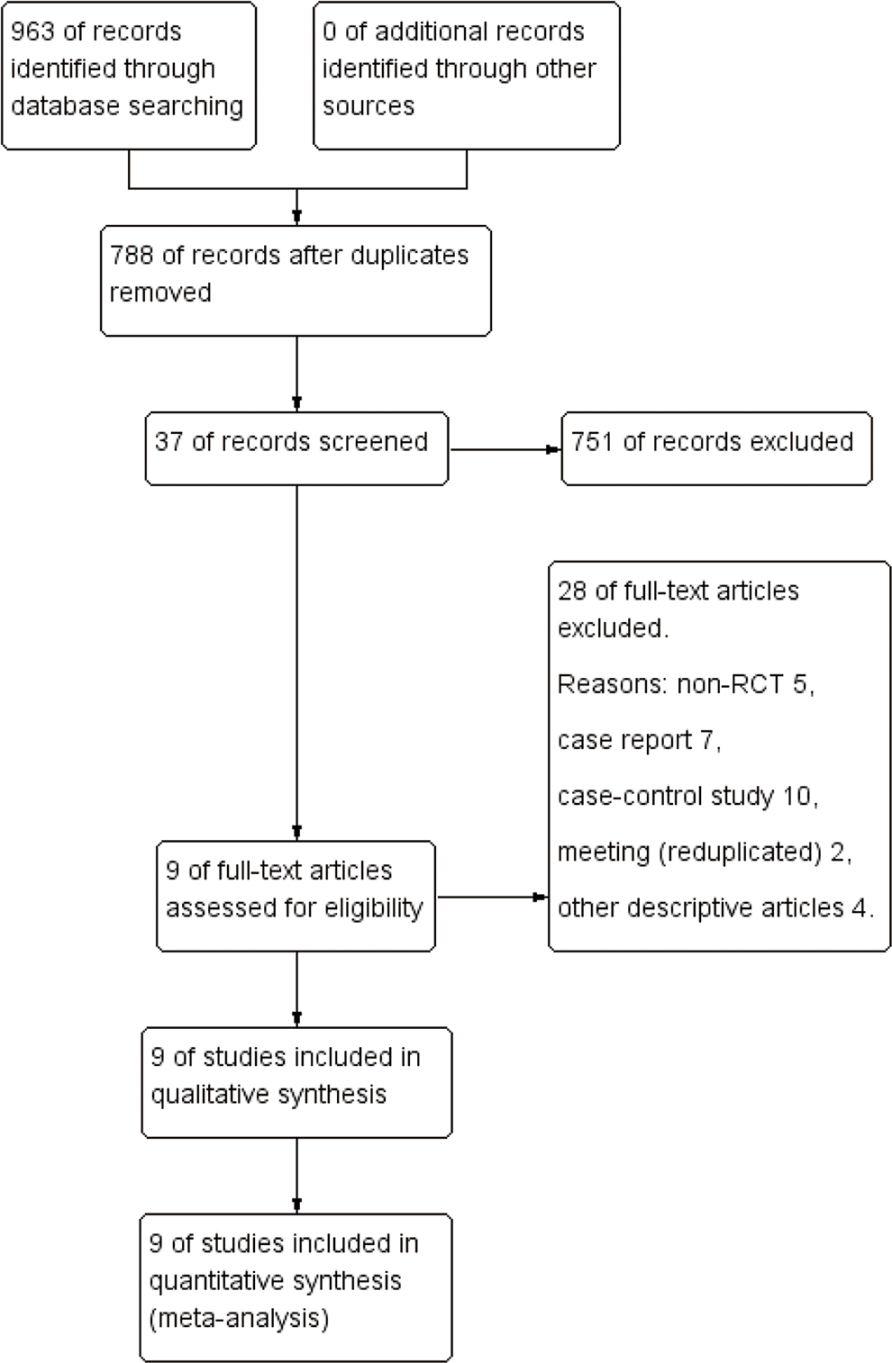
Flow diagram of the study selection process.

A total of 963 articles were identified in the database searches; no additional records were identified through other sources. After deleting any duplicates, 788 articles remained. At the initial screening stage, 751 articles were excluded based on their titles and abstracts because they did not meet the predefined inclusion criteria. The full texts of the 37 remaining articles were read. Of them, 28 articles were excluded for various reasons. At the end of the screening procedure, 9 studies remained [19–21,23–28]. The screening details are presented in a PRISMA flow diagram (Fig 1).

### Study characteristics and risk of bias

**Fig 2:**
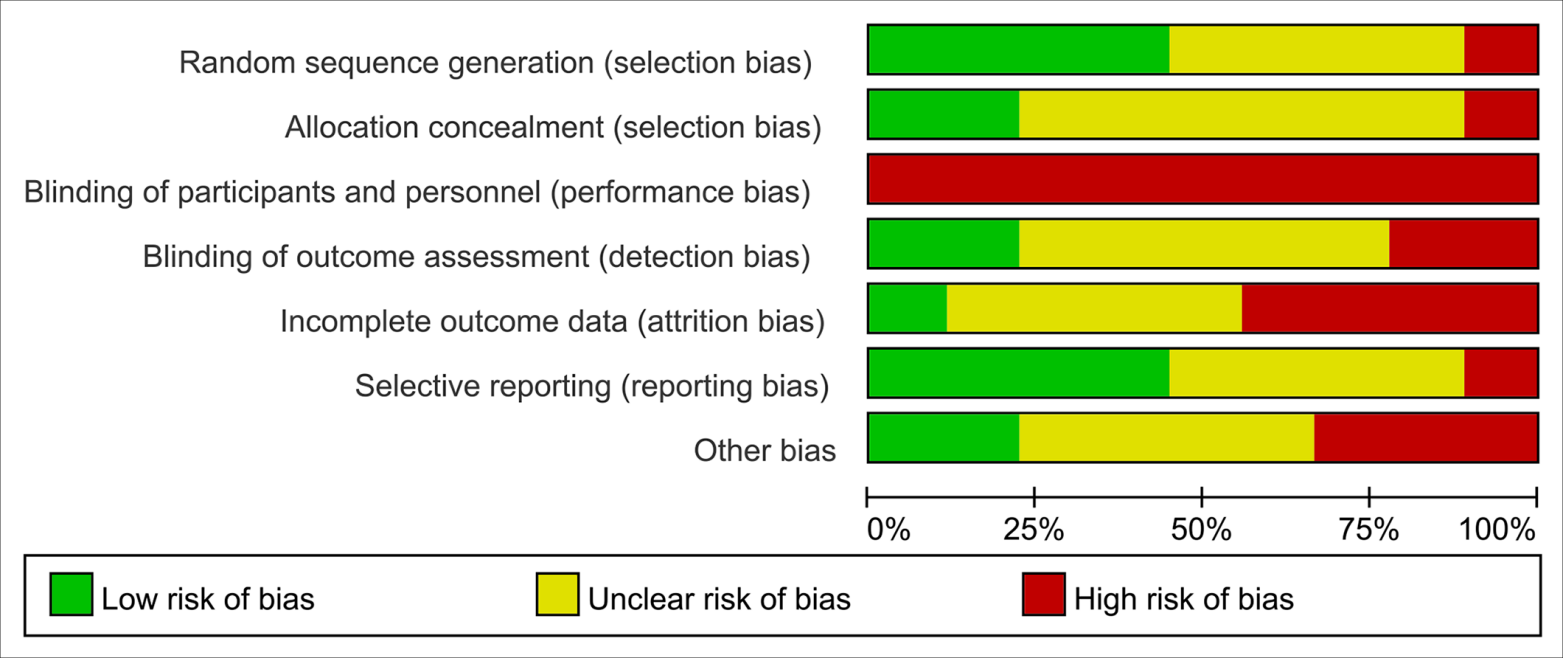
Risk of bias graph.

**Fig 3:**
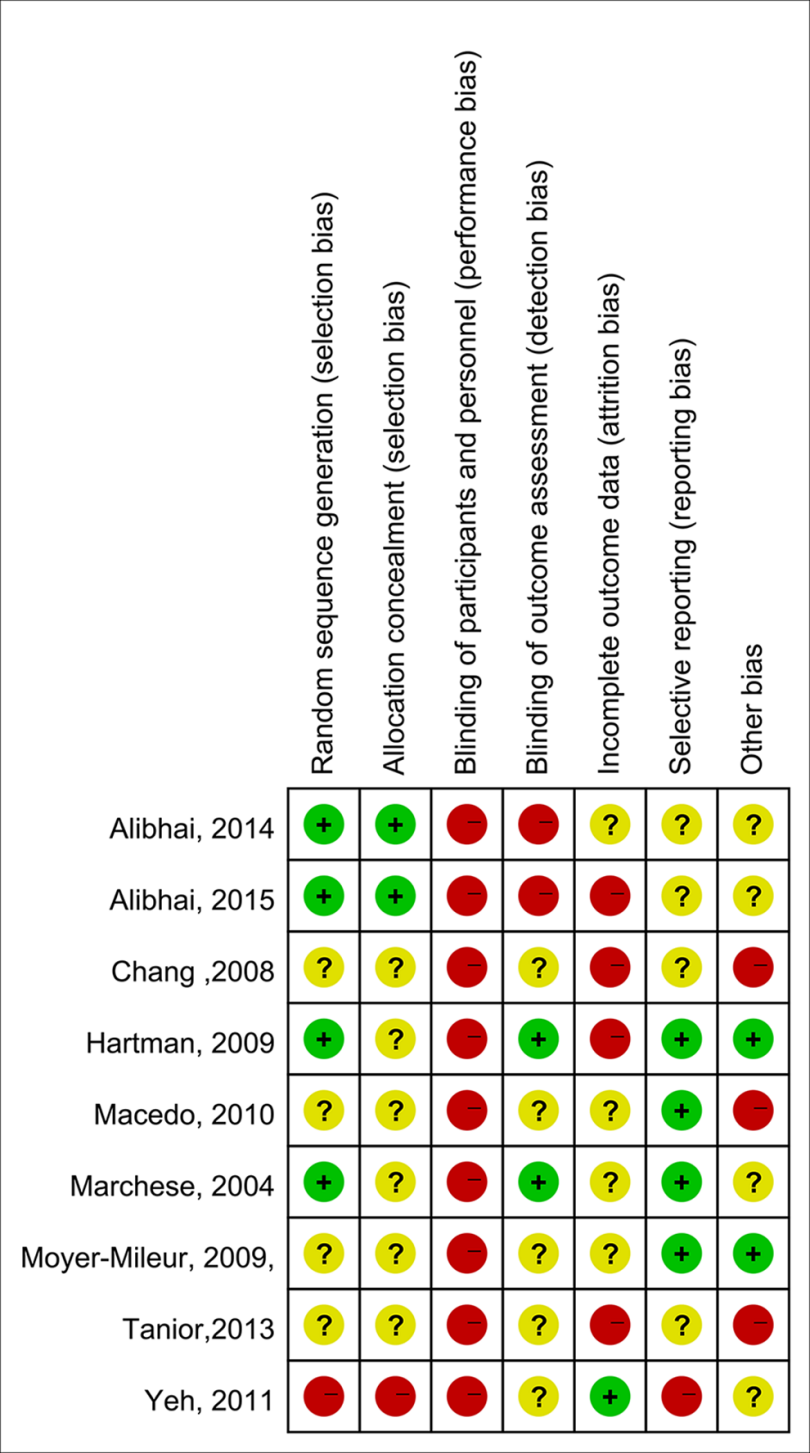
Risk of bias summary.

Nine trials were included in this meta-analysis [19–21,23–28]. Eight were RCTs, and 1 was a quasi-experimental design trial [28]. From these 9 studies, 314 participants were included in analysis (3 patients dropped out, 171 were males, 138 were females, and 2 were of unknown gender; 143 had AML, and 171 had ALL). The features of the included trials are summarized in Table 1. For a summary of each item associated with risk of bias, see Figs 2 and 3.

**Table 1:** Basic Characteristics of the Included Studies.

Study (author,year,type)	Participant characteristics	Intervention group	Comparison group	Outcomes measured	Follow-up times	Funding
Alibhai et al., 2015, RCT [19]	**Diagnosis:** AML	**Frequency:** 4–5 days/week	Usual care	**Self-reported measures:** EORTC-QLQC30,FACT-Fatigue anxiety and depression (HADS)	Baseline and 4–6 weeks	Grant from the Ontario Ministry of Health and Long Term Care
	**Department:** Cancer Center	**Intensity:** At an RPE of 3–6				
	**Timing:** Undergoing induction chemotherapy	**Time:** 30–60 min				
	**Participants:** N = 81 (57 intervention and 24 control)	**Type:** Mixed-modality exercise program (aerobic, resistance and flexibility training)				
				**Objective physical fitness:** VO2 peak/6 MWT/grip strength,MI/timed 10-chair stand test (lower body strength)		
	**Age:** •IG: 58±13.98 years					
	•CG: 52±15.8 years	**Duration:** Not reported				
	**Gender:** •IG: 14 males, 10 females					
	•CG: 30 males, 27 females					
	**Location:** Toronto, Canada					
Alibhai et al., 2014, RCT [20]	**Diagnosis:** AML	**Frequency:** 3–5 days/week	Usual care	**Self-reported measures:**EORTC-QLQC30 Fatigue (FACT-Fatigue), psychological distress (HADS)	Patient-reported outcomes at baseline and 3, 6, 9, 12, 18, and 24 weeks Fitness measures at baseline and 6, 12, and 24 weeks	Not reported
	**Department:** Princess Margaret Hospital	**Intensity:** A moderate–vigorous physical activity (MVPA) program				
	**Timing:** Completion of intensive chemotherapy or post-HSCT;confirmed CR					
		**Time:** 30 min				
	**Participants:** N = 38 (21 intervention and 17 control)	**Type:** Aerobic exercise, home-based exercise, strength training, flexibility exercise		**Objective physical fitness:** 6 MWT/grip strength/modified sit-and-reach/BMI/body fat/BP/HR		
	**Age:** •IG: 53.9±8.2 years	**Duration:** 12 weeks				
	•CG: 58.8±8.8 years					
	**Gender:** •IG: 10 males, 11 females					
	•CG: 7 males, 10 females					
	**Location:** Toronto, Canada					
De Macedo et al., 2010, RCT [23]	**Diagnosis:** ALL	**Frequency:** 2 times/day	Usual care	**Objective physical fitness:** Respiratory muscle strength (maximal inspiratory pressure and maximal expiratory pressure)	Baseline and 10 weeks	Not reported
	**Department:** Pediatric Oncology/ Hematology	**Intensity:** 30% of the PImax				
	**Timing:** During maintenance therapy	**Time:** 15 min				
	**Participants:** N = 14 (5 intervention and 9 control)	**Type:** Inspiratory muscle training program				
		**Duration:** 10 weeks				
	**Age:** children (8.3±2.6, 5~14 years)					
	•IG: 7.0 years					
	•CG: 9.0 years					
	**Gender:** 5 males, 9 females					
	**Location:** Brazil					
Tanir and Kuguoglu, 2013, RCT [21]	**Diagnosis:** ALL	**Frequency:** Active ROM exercise: 5 days/week, 3 sessions/day, 20 repetitions each session; leg exercises for muscle strengthening: 3 days/week, 3 times/day; aerobic exercises: 3 times/week	Usual care	**Self-reported measures:** PedQL 3.0 and 4.0	Baseline and 3 months	Not reported
	**Department:** Hematology-Oncology polyclinics					
	**Timing:** Remission			Objective physical fitness: 9 MWT; timed up and down stairs test, timed up and go test, isometric muscle strength		
	**Participants:** N = 41 (19 intervention and 21 control, 1 demise)	**Intensity:** Not reported				
		**Time:** Aerobic exercise: 0.5 h				
	**Age:** •IG: 10.21±1.51 years	**Type:** Home-based exercise program including active range of motion (ROM), leg muscle strengthening and aerobic exercises				
	•CG: 10.72±1.51 years					
	**Gender:** •IG: 15 males, 4 females					
	•CG: 9 males, 12 females	**Duration:** 3 months				
	**Location:** Istanbul, Turkey					
Yeh et al., 2011, quasi-experimental design trial [28]	**Diagnosis:** ALL	**Frequency:** 3 times/week	Usual care	**Self-reported measures:** Fatigue: PedQL Multidimensional Fatigue Scale	At baseline; once weekly during the 5-week intervention; at the end of the intervention; 1 month after the intervention	National Health Research Institutes, Taiwan National Science Council, Taiwan
	**Department:** Pediatric Oncology	**Intensity:** Increase in the patient’s HRR to a target of 40% to 60%				
	**Timing:** Maintenance chemotherapy					
	**Participants:** N = 24 (12 intervention and 10 control, 2 dropouts)	**Time:** 30 minutes				
		**Type:** Home-based aerobic exercise				
	**Age:** •IG: 11.0±3.56 years	**Duration:** 6 weeks				
	•CG: 12.5±3.86 years					
	**Gender:** • IG: 6 males, 4 females					
	• CG: 6 males, 6 females					
	**Location:** Taiwan, China					
Moyer-Mileur et al., 2009, RCT [25]	**Diagnosis:** Standard-risk ALL	**Frequency:** 3 times/week	Usual care	**Objective physical fitness:** Cardiorespiratory fitness: push-ups, sit-and-reach test, BMI, muscle mass, Flexibility: sit-and-reach distance test	Physical size at baseline and every 3 months Physical exercise at baseline and 6 and 12 months	Primary Children’s Medical Foundation
	**Department:** Pediatric Oncology	**Intensity:** A minimum of three 15- to 20-minute sessions of moderate-to-vigorous activity per week				
	**Timing:** Maintenance chemotherapy					
	**Participants:** N = 13 (6 intervention and 7 control)					
		**Time:** 15–20 minutes				
	**Age:** •IG: 7.2±0.7 years	**Type:** Home-based exercise and nutrition program				
	•CG: 5.9±0.7 years					
	**Gender:** 7 males, 6 females, 1 unknown (dropout)	**Duration:** 12 months				
	**Location:** USA					
Hartman et al., 2009, RCT [24]	**Diagnosis:** ALL	**Frequency:** Hand and leg function exercises: once/day; stretching and jumping exercises: 2 times/day	Usual care	**Objective physical fitness:** Body composition: BMI, Flexibility: passive ankle dorsiflexion	At diagnosis and 32 weeks, 1 year and 2 years after diagnosis, as well as at 3 years after diagnosis (1 year after cessation of treatment)	Not reported
	**Department:** Pediatric Oncology/Hematology, Pediatric Physiotherapy, Pediatric Endocrinology					
		**Intensity:** High-intensity				
	**Timing:** Undergoing chemotherapy	**Time:** Not reported				
	**Participants:** N = 51 (25 intervention and 26 control)	**Type:** Supervised and home-based exercise				
		**Duration:** 24 months				
	**Age:** •IG: 1.3~15.6 years					
	•CG: 1.7~17.1 years					
	**Gender:** •IG: 14 males, 11 females					
	•CG: 16 males, 10 females					
	**Location:** The Netherlands					
Chang et al., 2008, RCT [26]	**Diagnosis:** AML	**Frequency:** 5 times/week	Noninvasive routine care	**Self-reported measures:** Brief fatigue inventory 12 MWT Anxiety/depression status	Baseline and on days 7, 14 and 21	Not reported
	**Timing:** Undergoing chemotherapy	**Intensity:** Increase in heart rate to the target heart rate (30 beats/min above the resting heart rate)				
	**Department:** Hematology inpatient ward					
	**Participants:** N = 24 (11 intervention, 11 control, and 2 dropouts)					
		**Time:** 12 min/day				
	**Age:** •IG: 49.4±15.3 years	**Type:** Aerobic exercise (walking program)				
	•CG: 53.3±13.6 years	**Duration:** 3 weeks				
	**Gender:** •IG: 8 males, 3 females					
	•CG: 4 males, 7 females					
	**Location:** Taiwan, China					
Marchese et al., 2004, RCT [27]	**Diagnosis:** ALL	**Frequency:** Ankle dorsiflexion stretching exercises: 30 seconds/session, 5 days/week; bilateral lower extremity strengthening exercises: 3 sets of 10 repetitions, 3 days/week; aerobic exercise: once/day	Usual care	**Self-reported measures:** PedQL version 3.0	Endpoint measurements at baseline and 4 months	Grant from The Children's Hospital of Philadelphia
	**Timing:** During maintenance therapy					
	**Department:** Pediatric Rehabilitation, Pediatric Oncology, Pediatric Physiotherapy					
				**Objective physical fitness:** 9 MWT, Timed up and down stairs test, Knee extension strength and ankle dorsiflexion strength, Ankle dorsiflexion range of motion		
	**Participants:** N = 28 (13 intervention and 15 control)	**Intensity:** Not reported				
		**Type:** 5 hospital-based physiotherapy sessions, ankle dorsiflexion stretching exercises, bilateral lower extremity strengthening exercises, aerobic exercise				
	**Age:** •IG: 7.6, 4.3~10.6 years					
	•CG: 8.6, 5.1~15.8 years					
	**Gender:** •IG: 12 males, 13 females	**Time:** 20–60 minutes				
	•CG: 8 males, 5 females	**Duration:** 4 months				
	**Location:** USA					

AML, acute myelocytic leukemia; ALL, acute lymphoblastic leukemia; IG, intervention group; CG, control group; BMI, body mass index; 6 MWT, 6-minute run-walk test; 9 MWT, 9-minute run-walk test; 12 MWT, 12-minute run-walk test; EORTC QLQ-C30, European Organization for Research and Treatment of Cancer core 30-item questionnaire; FACT-F, Functional Assessment of Cancer Therapy-Fatigue; HADS, Hospital Anxiety and Depression Scale, BFI, Brief Fatigue Inventory; RPE, rating of perceived exertion; HRR, heart rate reserve; PImax, maximum inspiratory pressure.

### Meta-analysis of outcome measures

#### Cardiorespiratory fitness

**Fig 4:**
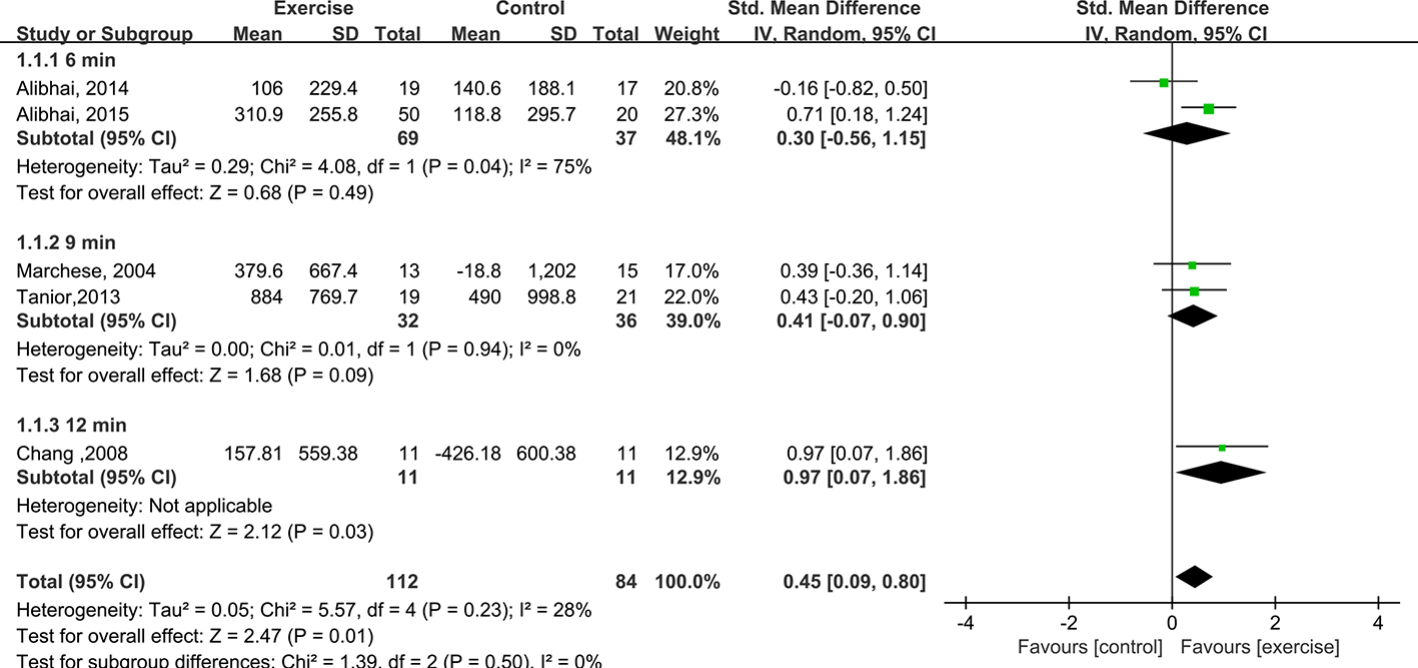
Forest plot comparing the changes in cardiorespiratory fitness between the exercise and control groups.

In five included trials (n = 196), cardiorespiratory fitness was assessed via run-walk tests, with varying durations between the trials [19–21,26,27]. Subgroup analyses were conducted on the 6-min, 9-min and 12-min run-walk test subgroups. The cardiorespiratory fitness of the 6- and 9-min subgroups did not significantly differ between the exercise and control interventions; however, the 12-min subgroup showed a significant improvement in cardiorespiratory fitness for the exercise intervention compared to the control intervention. The aggregated results of the five studies revealed a significant difference in cardiovascular fitness between the interventions (SMD = 0.45, 95% CI: 0.09 to 0.80, P value = 0.01, P for heterogeneity = 0.23, I^2^ = 28%). The details are presented in Fig 4.

#### Muscle strength

**Fig 5:**
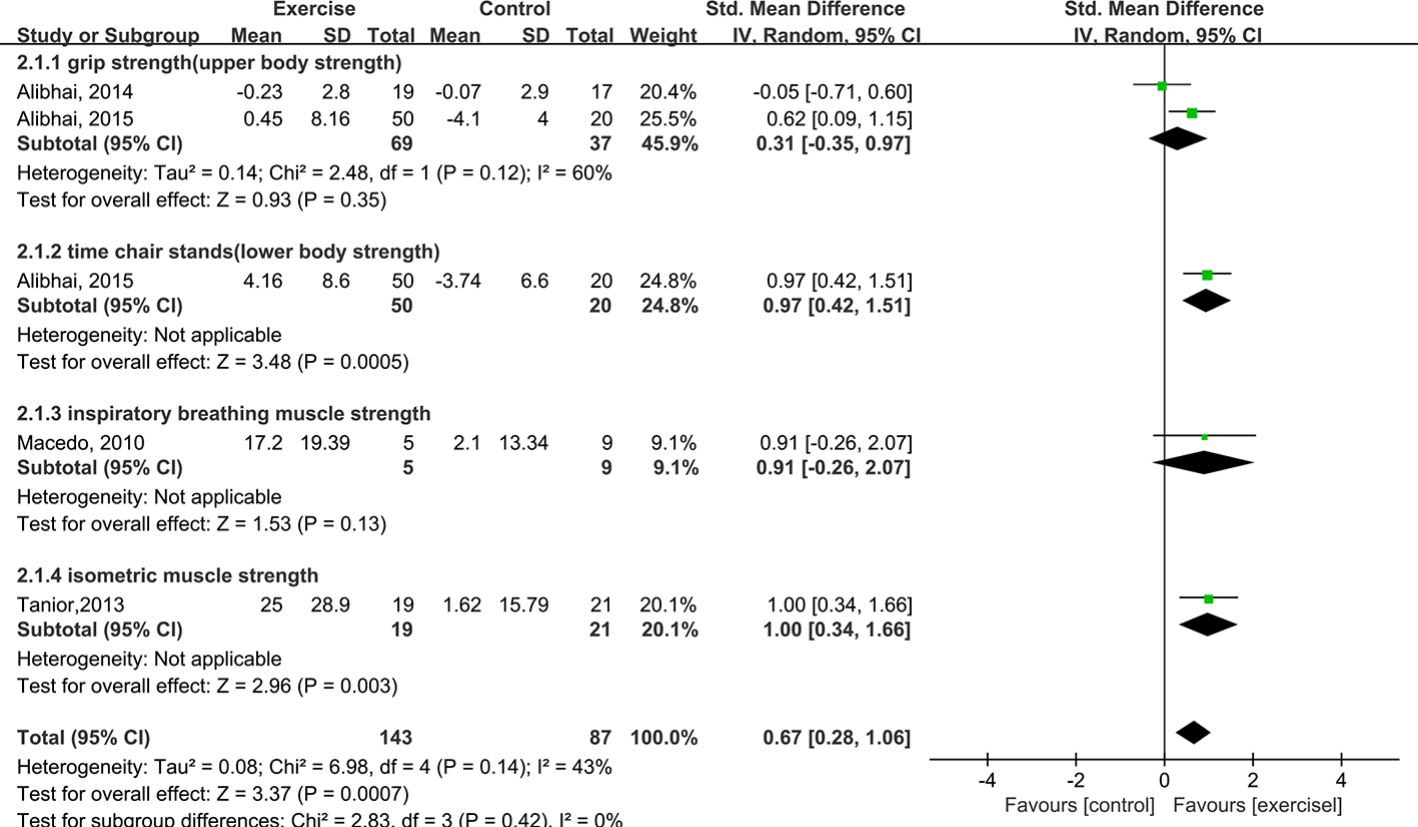
Forest plot comparing the changes in muscle strength between the exercise and control groups.

Four studies (n = 160) measured muscle strength in AL patients [19–21,23]. Muscle strength was assessed via different measurements for different muscle groups. Alibhai et al. [19,20] measured grip strength using a Jamar dynamometer and lower body strength via the timed 10-chair stand test. Tanir and Kuguoglu [21] measured isometric muscle strength using a dynamometer. De Macedo et al. [23] measured respiratory muscle strength based on maximum inspiratory and expiratory pressure. The aggregated results of these studies showed a significant difference in muscle strength between the intervention and control groups, with an SMD of 0.67 (95% CI: 0.28 to 1.06, P value = 0.0007, P for heterogeneity = 0.14, I^2^ = 43%) (Fig 5).

#### Fatigue, anxiety and depression

**Fig 6:**
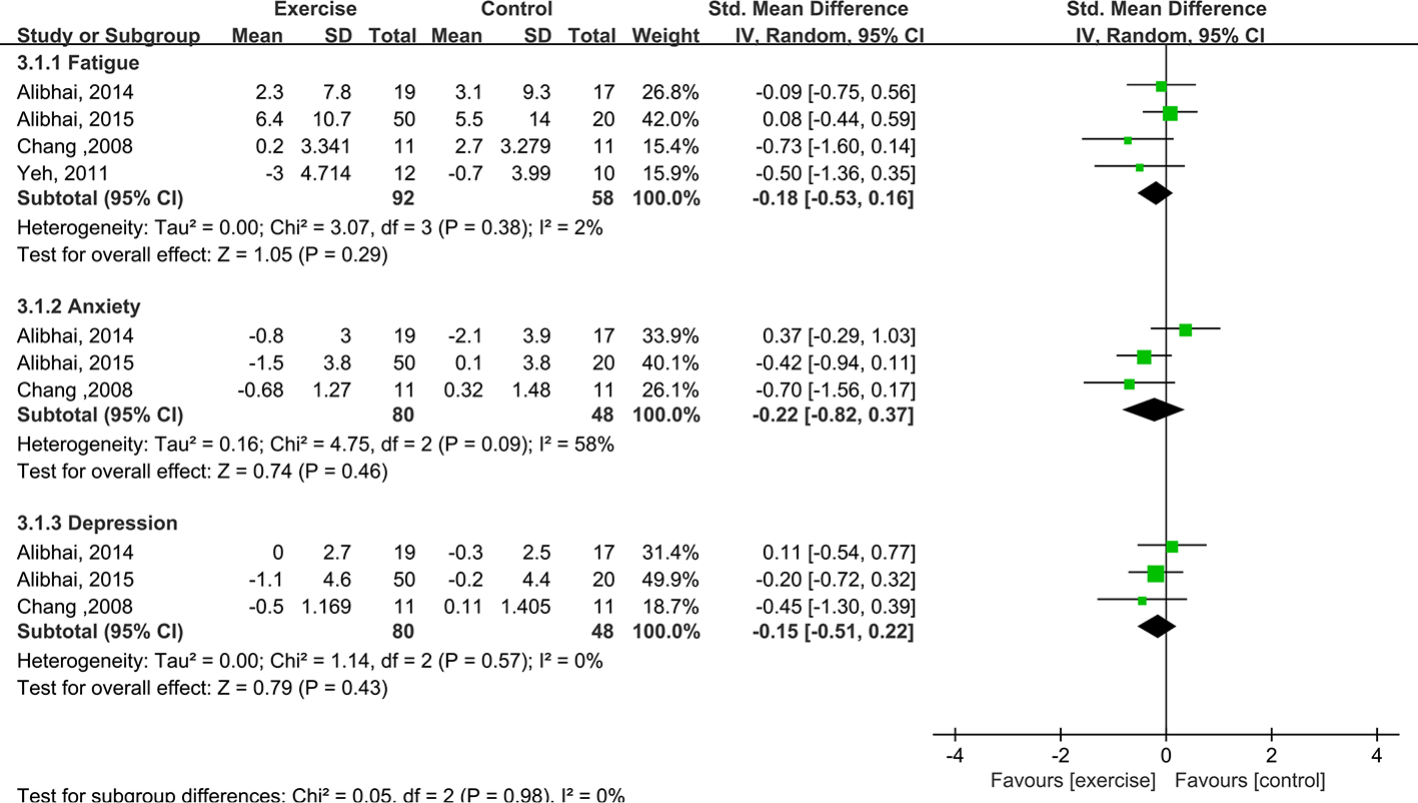
Forest plot comparing the changes in fatigue, anxiety and depression between exercise and control groups.

Four studies (n = 160) [19,20,26,28] provided data on fatigue measurements. Fatigue was assessed by Alibhai et al. [19,20] using the Functional Assessment of Cancer Therapy-Fatigue subscale (FACT-Fatigue), by Chang et al. [26] using the Brief Fatigue Inventory (BFI), and by Yeh et al. [28] using the PedQL Multidimensional Fatigue Scale. The aggregated results of these studies showed no significant difference in fatigue between the groups (SMD = -0.18, 95% CI: -0.53 to 0.16, P value = 0.28, P for heterogeneity = 0.38, I^2^ = 2%). Anxiety and depression were assessed by Alibhai et al. [19,20] using the Hospital Anxiety and Depression Scale and by Chang et al. [26] using the depression and anxiety subscales of the Profile of Mood States Short Form. The results revealed non-significant differences in anxiety and depression between the groups (anxiety: SMD = -0.22, 95% CI: -0.82 to 0.37, P value = 0.46, P for heterogeneity = 0.09, I^2^ = 58%; depression: SMD = -0.15, 95% CI: -0.51 to 0.22, P value = 0.28, P for heterogeneity = 0.57, I^2^ = 0%) (Fig 6).

**Fig 7:**
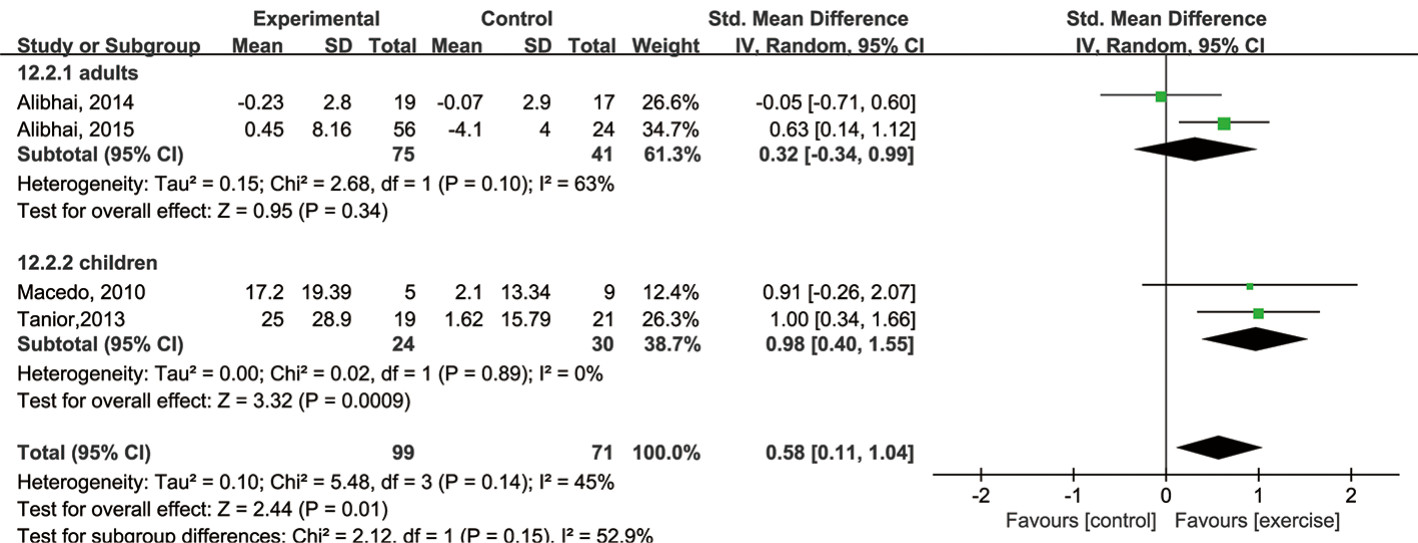
Forest plot of subgroup analyses comparing changes in muscle strength among adults and children.

We next performed subgroup analyses of adults and children. Among the adults, there were non-significant differences in cardiorespiratory fitness, muscle strength, and fatigue between the exercise and control groups. Among the children, there were non-significant differences in cardiorespiratory fitness and fatigue but a significant difference in muscle strength between the two groups (SMD = 0.98, 95% CI: 0.40 to 1.55, P value = 0.0009, P for heterogeneity = 0.89, I^2^ = 0%) (Fig 7). In addition, subgroup analyses based on induction therapy and post-remission therapy were performed. For induction therapy, there was a non-significant difference in fatigue but significant differences in cardiorespiratory fitness and muscle strength between the exercise and control groups (cardiorespiratory fitness: SMD = 0.78, 95% CI: 0.32 to 1.24, P value = 0.0009, P for heterogeneity = 0.63, I^2^ = 0%; muscle strength: SMD = 0.63, 95% CI: 0.14 to 1.12, P value = 0.01, heterogeneity: not applicable). For post-remission therapy, there were non-significant differences in cardiorespiratory fitness, muscle strength, and fatigue between the exercise and control groups. Thus, significant differences were found in muscle strength among children with AL undergoing induction therapy and in cardiorespiratory fitness among AL patients undergoing induction therapy regardless of age.

#### Other outcomes

Functional mobility was measured using the “timed up and down stairs test” and the “timed up and go test” [21,27]. The aggregated results of the “timed up and down stairs test” showed a significant difference between the exercise and control groups, with an MD of -1.52 (95% CI: -2.38 to -0.65, P value = 0.0006, P for heterogeneity = 0.20, I^2^ = 39%). The results of the “timed up and go test” also revealed a significant difference between the exercise and control groups (SMD = -1.15, 95% CI: -1.83 to 0.48, P value = 0.0008). Alibhai et al. [19,20] assessed overall health-related QOL using the European Organization for Research and Treatment of Cancer (EORTC) questionnaire for cancer patients (the EORTC-QLQC30), and Marchese et al. [27] assessed QOL using the general Pediatric Quality of Life Inventory^TM^ Measurement Model (PedQL) 3.0 and the cancer PedQL. However, because the outcome definitions of these instruments differed, we did not pool these data. The aggregated results of Alibhai et al. [19,20] showed a non-significant difference in overall health-related QOL between the exercise and control groups (SMD = -7.61, 95% CI: -16.34 to 1.11, P value = 0.09, P for heterogeneity = 0.31, I^2^ = 3%). The cancer and general PedQL scores reported by Marchese et al. [27] also indicated a non-significant difference between the groups. Marchese et al. [27] and Tanir and Kuguoglu [21] measured blood physiological parameters, including the hemoglobin level and hematocrit. They reported a non-significant difference in the SMD in the hemoglobin level between the exercise and control groups (SMD = -0.29, 95% CI: -0.19 to 0.77, P value = 0.23, P for heterogeneity = 0.80, I^2^ = 0%). In addition, differences in BMI, which was assessed to evaluate body composition, between the two groups were studied in two trials [24,25]. The pooled results of these studies showed a non-significant difference in BMI between the exercise and control groups (SMD = 0.60, 95% CI: -0.25 to 1.44, P value = 0.17, P for heterogeneity = 0.16, I^2^ = 51%).

## Discussion

This meta-analysis provides a comprehensive summary of the current evidence supporting the efficacy of exercise in AL patients. The results suggest that exercise significantly improves cardiorespiratory fitness and muscle strength. Functional mobility, which was measured using the “timed up and down stairs test” and the “timed up and go test”, also showed significant improvement in the exercise group compared to the control group. However, no improvements in fatigue, anxiety, depression, QOL, the hemoglobin level, hematocrit or BMI were observed in the exercise group compared to the control group.

Theoretical evidence of the effects of exercise has emerged. Winningham’s Psychobiological-Entropy Model, a theoretical framework for cancer-related fatigue, proposes that a balance between rest and exercise is vitally important for reducing fatigue but that an imbalance could lead to functional deterioration [29]. In addition, the National Comprehensive Cancer Network has stated that exercise reduces cancer-related fatigue by improving the functional capacity, thereby reducing effort and perceived fatigue [30]. Dimeo [31] has also noted that the prevailing recommendations that patients rest and avoid intensive exercise are likely to be counterproductive and lead to muscle wasting, reduced cardiorespiratory fitness and increased fatigue. Several biological mechanisms of the effects of exercise on patients with solid tumors have been reported. Mock [32] has suggested that endorphins, a class of depressants, are secreted via pituitary gland stimulation resulting from exercise, thereby improving the responsiveness of the central nervous system and the body’s tolerance to strong stimulation. Simultaneously, the nervous system produced microstimulation during exercise, which relieves muscle tension, anxiety and depression. In addition, exercise enhance metabolism to promote the clearance of metabolic waste and accumulated adrenaline. Exercise also promotes blood circulation, resulting in improved organ function. Thus, exercise has beneficial effects on patients’ cardiorespiratory fitness, muscle strength, fatigue, and negative moods.

This is the first meta-analysis to summarize the efficacy of exercise for individuals with AL based on document retrieval. Our results are similar to those of the most recent SR [16]. In detail, that review included five studies demonstrating that exercise interventions appear to be safe and feasible for individuals with AML, although the evidence was insufficient to support the benefits of exercise for chronic myelocytic leukemia patients. However, the conclusions of that SR were limited because of variation in study types, small sample sizes and lack of meta-analysis. Only two studies in that SR were included in our meta-analysis. Other reviews have focused on hematologic malignancies. Bergenthal et al. [3] conducted a Cochrane review evaluating the safety, feasibility and efficacy of aerobic exercise for adults with hematologic malignancies. Liu et al. [14] reviewed ten studies (three RCTs and seven non-RCTs) that examined the effects of exercise on children and adults suffering from hematologic cancers. Further, Wolin et al. [4] investigated the effects of exercise on adult and pediatric survivors of hematologic cancer (including five RCTs and eight non-RCTs). Only 3 RCTs [24,26,27] mentioned in these reviews were included in our meta-analysis. In contrast to the previous reviews, the present meta-analysis showed that exercise significantly improved cardiorespiratory fitness and muscle strength, and these findings are consistent with those of Bergenthal et al. [3]. In this meta-analysis, all studies that assessed fatigue, anxiety and depression showed positive trends of improvements in fatigue and mood due to exercise. However, the aggregated results did not demonstrate a significant difference in fatigue or anxiety between the exercise and control groups, whereas Bergenthal’s study did show significant differences in these parameters.

In addition, subgroup analyses according to age and treatment stage were conducted to determine the optimal exercise parameters for individuals with AL. These analyses revealed that exercise significantly improved muscle strength in children with AL undergoing induction therapy and cardiorespiratory fitness in AL patients of any age undergoing induction therapy. Additional evidence is needed to validate and expand upon these findings.

To determine the most effective type, intensity and duration of exercise, further trials with more participants and rigorous study designs are needed. The comparability of the study data could also be enhanced by standardizing the measurement instruments. We found that most studies lacked objective outcomes, such as BMI, the hemoglobin level, and peak VO_2_, as well as safety and feasibility assessments, which would have resulted in more reliable and convincing evidence for clinical applications. In the future, interdisciplinary teams, including basic laboratory scientists, rehabilitation specialists and physical therapists, are expected to participate in further studies.

### Limitations

This meta-analysis has some limitations. One major limitation is the small number of included studies, which restricted our ability to summarize the exercise data for individuals with AL with different characteristics and to examine the efficacy of exercise by conducting subgroup analyses according to the frequency, intensity, time, and type of exercise. For the same reason, a funnel plot could not be generated to evaluate publication bias. Moreover, as an intervention, exercise is typically evaluated by researchers themselves by performing trials because there is no manufacturer or company that can probe for missing data. These factors and missing data may have impacted our results and led to bias.

## Conclusion

In summary, the positive findings of this meta-analysis suggest that exercise has beneficial effects on cardiorespiratory fitness, muscle strength and functional ability. No significant exercise-induced improvements in fatigue, anxiety, depression or QOL were found. AL patients need to exercise in order to counteract side effects (fatigue and myasthenia) with caution due to risk of bleeding. Considering this dilemma, it is necessary to conduct further research on this population. We hope to attract more researchers to this topic so that additional evidence of the efficacy of exercise for AL patients can be obtained. To date, due to the shortage of relevant studies, there is insufficient evidence available to assess the safety, feasibility and efficacy of exercise programs for AL patients. Therefore, high-quality RCTs examining exercise interventions are imperative for this population.
